# Quality characteristics, fatty acid profiles, flavor compounds and eating quality of cull sow meat in comparison with commercial pork

**DOI:** 10.5713/ajas.19.0262

**Published:** 2019-08-03

**Authors:** Van Ba Hoa, Soo-Hyun Cho, Pil-Nam Seong, Sun-Moon Kang, Yun-Seok Kim, Sung-Sil Moon, Yong-Min Choi, Jin-Hyoung Kim, Kuk-Hwan Seol

**Affiliations:** 1 National Institute of Animal Science, Rural Development Administration, Wanju 55365, Korea; 2 Sunjin Meat Research Center, Ansung 17532, Korea; 3 National Institute of Agricultural Sciences, RDA, Wanju 55365, Korea

**Keywords:** Cull Sow Meat, Finishing Pig Meat, Flavor Compound, Sensory Quality

## Abstract

**Objective:**

Although the slaughter of cull sows (CS) for human consumption and meat products processing appears quite common throughout the world, relatively limited scientific information regarding the meat quality parameters of this pork type is available. The present study aimed at providing the technological quality characteristics and eating quality of CS meat, and comparing with those of commercial pork.

**Methods:**

*Longissimus thoracis et lumborum* muscle samples of CS and finisher pigs (FP) at 24 h *postmortem* were collected and used for investigation of the meat quality traits (pH, color, shear force, cooking loss, water holding capacity), fatty acids, flavor compounds and sensory characteristics.

**Results:**

The CS meat had significantly higher moisture content (p = 0.0312) and water holding capacity (p = 0.0213) together with lower cooking loss (p = 0.0366) compared to the FP meat. The CS meat also exhibited higher (p = 0.0409) contents of unsaturated fatty acids, especially polyunsaturated fatty acids (PUFA, p = 0.0213) and more desirable PUFA/total saturated fatty acids ratio (p = 0.0438) compared to the FP meat. A total of 56 flavor compounds were identified, amongst the amount of 16 compounds differed significantly between the two pork groups. Most of the PUFA-derived flavor compounds (e.g., hexanal, benzaldehyde, and hydrocarbons) showed higher amounts in the CS meat. While, 3-(methylthio)-propanal and 4-methylthiazole associated with pleasant aromas (meaty and roast odor notes) were only found in the FP meat. Furthermore, no differences were reported by panelists for flavor, juiciness, tenderness, and acceptability scores between the two pork groups studied.

**Conclusion:**

The sow meat exhibited better technological quality and its eating quality could be comparable to the commercial pork. This study provides meat processors and traders with valuably scientific information which may help to improve the utilization and consumption level of sow meat.

## INTRODUCTION

It is estimated that there are millions of sows being reared and exploited in breeding farms worldwide, and yearly thousands of these sows are removed from these breeding farms due to ageing and reproduction loss etc. [1,2]. The slaughter of cull sows (CS) for human consumption and meat products processing is quite common throughout the world [2]. To date, there are only a few studies investigating the effects of on-farm factors (e.g., rearing environment) on the quality characteristics of sow meat as well as its processed products (e.g., cured bacon marinated meat) [3,4]. Pork is one of the most consumed meat types worldwide, and its quality is defined as a set of properties consisting of attributes such as appearance, color, flavor, texture, juiciness, water holding capacity (WHC) and odors [5]. However, the meat derived from CS is associated with some problems such as darker color, less tenderness and larger muscle size due to heavier body weight, which hinder its usage for whole muscle meat products [3]. Sow meat therefore is usually used for production of comminuted products such as fresh sausages and meatballs etc.

To the best of our knowledge, little scientific information regarding the quality characteristics of sow meat is available. Also, no studies have been conducted to evaluate the consumer’s perception of sow meat or to compare its quality, fatty acids composition and flavor compounds with those of pork from growing-finishing pigs (FP). Because of a large number of sows are removed from breeding herds every day worldwide, investigation of quality characteristics is necessary to improve the consumption and utilization level as well as to increase the economic value of the sow meat. To reach this aim, more studies should be conducted to provide consumers and processors with further information on its technological quality attributes, consumer’s perception and flavor characteristics. Furthermore, the most important aspect of meat quality is eating quality, usually defined as scores given by taste panelists for sensorial traits such as flavor and tenderness [6], however, the eating quality such as flavor attribute of CS meat has not been extensively studied. Especially, till now the composition of volatile flavor compounds produced in sow meat during cooking have not been identified and quantified.

Thus, the objective of this study was to provide and com pare the technological quality characteristics (e.g., pH, color, WHC, cooking loss) eating quality (flavor, juiciness, and tenderness) and volatile flavor compounds between meats obtained from CS and FP. The findings of this study could be valuable for pork processors and traders to determine suitable solutions for improving the utilization, processing and consumption level as well as to increase the economic value of sow meat.

## MATERIALS AND METHODS

### Animals and samples preparation

Crossbred ([Landrace×Yorkshire]♀×Duroc ♂) FP at ages of about 175 to 185 days with average body weights of 110 to 115 kg collected from commercial pig farms, and Berkshire CS at ages from 1,085 to 1,421 days old with average body weights of approximately 230 to 240 kg collected from breeding farms in Korea were used in the present investigation. The pigs were transported from the farms to a commercial slaughterhouse of National Institute of Animal Science (Korea) with the transporting time of about 1 to 2 h. After arriving, the pigs were laired in pens for 6 to 8 h with full access to water but fasted from feeds. The pigs slaughter was done under the commercial slaughtering process as shown in our previous study [7]. After chilling for 24 h at (2°C±2°C), the carcasses were transferred to a cutting room where the *longissimus thoracis et lumborum* (LTL) muscles were collected from the left carcasses side and used for the meat quality analyses. The muscle samples (n = 10 each) were then trimmed off all visual fats and connective tissues, made into sub-samples depending analyses. The pH, instrumental color, cooking loss, shear force, WHC, and proximate composition were determined on the same sampling day while, the samples used for fatty acids, flavor compounds and sensory evaluation were vacuum-packaged and stored at −20°C until use.

### pH measurement

The pH values were determined on the pork samples using a pH*K 21 (NWK-Technology GmbH, Kaufering, Germany) equipped with a stainless steel and solid-state probe. Before using, the pH meter was calibrated with pH 4.0 and 7.0 standards (NWK Technology., Germany) according to the manufacturer’s instruction, and then the probe was inserted deeply into the muscle tissue. The pH values of each sample were the average of the three readings.

### Instrumental color measurement

Approximately 30 min after blooming, the color attributes were determined on 3 different locations of the freshly-cut surface of each sample using a Minolta Chroma Meter CR-400 (Minolta Camera Co., Ltd., Osaka, Japan). Before using, the device was standardized with a white plate (Y = 86.3, X = 0.3165, and y = 0.3242). Color was expressed according to the Commission International de l’Eclairage (CIE) system and reported as CIE L* (lightness), CIE a* (redness), CIE b* (yellowness), Chroma and hue angle (h°). In which the Chroma and hue angle were calculated as (a*^2^+b*^2^)^0.5^ and tan^−1^ (b*/a*), respectively.

### Proximate composition

The proximate compositions such as moisture, protein and fat contents were determined using a Food Scan Lab 78810 (Foss Tecator Co., Ltd., Hillerod, Denmark), as described in previous study [8]. Briefly, after grinding, about 200 g of ground meat sample (each) was placed onto the instrument’s round sample dish and then loaded into the instrument’s sample chamber. Each sample was determined in triplicates.

### Cooking loss and Warner-Bratzler shear force value measurements

The cooking loss and Warner-Bratzler shear force (WBSF) were measured on a same 3.0-cm thick steak (approximately 200 g) of each muscle sample, as described in our previous work [7]. Briefly, the samples were placed in plastic bags, sealed with double clips and put in a pre-heated 72°C water bath, and kept until their core temperature had reached 70°C. The core temperature of samples was monitored using a copper-constantan thermocouple attached to a Thermo recorder (Model TR-71U; T & D Corp., Tokyo, Japan). The cooked samples were then immediately cooled for 30 min in a circulatory water bath. After removing the plastic bags and absorbed with wiping papers to remove the surface water, the samples were re-weighed to determine the cooking loss.

After determining the cooking loss, these samples were used for the WBSF analysis. Particularly, 5 representative cores with an average diameter of 0.5 inches and length of about 2 cm were removed parallel to the muscle fiber direction of each sample using a 0.5-inch metal corer. The WBSF values were obtained by completely cutting the cores in an Instron Universal Testing Machine (Model 4465, Instron Corp, High Wycombe, UK) using a crosshead speed of 200 mm/min and a 40 N load cell.

### Water holding capacity

The WHC of the pork samples was determined using the protocol as described in our previous study [7] and Han et al [9]. Particularly, after grinding with a mini grinder, approximately 0.5 g of each sample was taken and placed in a 2 mL ultra-centrifugal filter unit, inserted into an ultra-centrifugal filter device (Millipore Corp., Bedford, MA, USA), and then heated in an 80°C pre-heated water bath for 20 min. The samples were then centrifuged at 2,000×*g* for 10 min at 4°C after cooling at room temperature for 10 min. Thereafter, the weight of ultra-centrifugal filter unit containing the cooked sample was recorded to determine the water loss. Simultaneously, the moisture content of each fresh sample obtained from the proximate composition as described above was also used to determine its WHC. Each sample was done in duplicates and the WHC percentage was calculated as a ratio of moisture to the water loss.

### Fatty acid profiles

Extraction and separation of fatty acids content in pork samples were done following the protocol as described in our previous study [7]. The fatty acids were determined using a gas chromatography/flame ionization detector system (Model Star 3600, Varian Technologies, Palo Alto, CA, USA). Fatty acids were separated on an Omegawax 205 fused silica bond capillary column (30 m×0.25 mm×0.25 μm film thickness; Supelco, Bellefonte, PA, USA) at a split ratio of 100:1. Nitrogen was the carrier gas in constant pressure mode at 16.0 psi and a flow rate of 1.0 mL/min. An aliquot of 2 μL of each sample was injected into the injection port, and the temperatures of injector and detector were set at 250°C and 260°C respectively. The oven temperature was held at 50°C for 1 min, and then raised to 200°C at a rate of 25°C/min, and further increased to 230°C at a rate of 5°C per min. The free fatty acids in samples were identified by comparing their retention times with those obtained from standard fatty acids. The results were expressed as relative percent (%) of total fatty acids based on total peak area.

### Sensory evaluation

The sample preparations and sensory evaluation were performed using the method of Seong et al [8] with minor modifications. Briefly, each sample from each pork group was tested by six panels allocated in randomized block arrangement. The panels consisted of 6 trained members who were staffs at the Animal Products Processing Division of National Institute of Animal Science. Each session had six panelists; each panelist evaluated six samples, and two sessions per day (10:30 am and 15:00 pm) were performed. Prior to use, the vacuum-packed samples were removed from a freezer (−20°C) and defrosted for 2 h in a 4°C cooler. For each sample, 7 representative slices (50 mm×50 mm×4 mm) were prepared parallel to fiber direction, in which 1 slice was used for the sensorial color evaluation. For the sensorial color evaluation, the freshly-cut slices were evaluated after 30 min cutting (blooming), each the slice was passed through to all the panelists. The remaining 6 slices/sample were cooked on an open tin-coated grill for about 2 min and turned 30 s intervals. The cooking temperature was monitored using an infrared thermometer and was maintained at around 180°C. One set of grill was used where the grill was set to cook six slices of samples. Immediately after cooking, the samples were placed on individual dishes and served to the panelists. The panelists then handled the cooked samples with an approved odorless plastic fork and tasted for flavor, juiciness, tenderness and overall acceptability using a 7-point scale (7 = extremely like; 6 = like very much; 5 = like moderately; 4 = neither like nor dislike; 3 = dislike moderately; 2 = dislike very much; and 1 = dislike extremely) as described by Meilgaard et al [10]. The panelists were asked to refresh their palate with drinking water and unsalted crackers between samples. All sensory sessions were carried out in the sensory panel booth room equipped with white lighting.

### Volatile flavor compounds

The volatile flavor compounds in cooked pork samples were determined using our previously standardized method [11] with minor modifications. Briefly, after cooking (the cooking method and condition was same as those in the sensory evaluation), 1.0 g of each sample was taken and placed into a 20-mL headspace vial and sealed with polytetrafluoroethylene-faced silicone septum for extraction. The extraction of volatile flavor compounds was done using solid-phase micro-extraction (SPME). A SPME device containing carboxen–polydimethylsiloxane (75 μm) fibre (Supelco, USA) was used for extraction of the compounds. The extraction, absorption, desorption of the flavor compounds was done using a fully automated SPME sample preparation instrument (Model: AOC-5000 Plus) connected to gas chromatography (GC) (Model: 7890B GC) with mass spectrophotometry (Model: 5977B MSD, Agilent Technologies, Santa Clara, CA, USA). The extraction was carried out at 65°C and agitated at 250×rpm for 60 min. The gas chromatography-mass spectrophotometry conditions used for separation of the volatiles were same as those mentioned in the above cited literature. Identifications of volatiles were performed by: i) comparing their mass spectra with those already present in the Wiley registry of mass spectral data (Agilent Technologies, USA), and ii) by comparing their retention times with those of external standards. Approximated quantities of the volatile compounds were quantified by comparison of their peak areas with that of the 2-methyl-3-heptanone internal standard obtained from the total ion chromatogram using a response factor of 1.

### Statistical analysis

The obtained data was analyzed using a Statistic Analysis System (SAS) package (SAS Institute, Cary, NC, USA, 2007). Means and standard errors were calculated for all the analytic items using the means procedure of the SAS. The data were then analyzed by using the general linear model procedure. Differences between pairs of means were measured using Duncan’s multiple range test. Significance was defined at p<0.05.

## RESULTS AND DISCUSSION

The final purposes of sows and commercial pig production as well as their raising conditions (e.g., feed and feeding regimes) are completely different. The CS-derived meat, however, is commonly consumed and utilized in meat products worldwide [2]. In the present investigation, we aimed at providing the technological quality traits, fatty acids content, flavor compounds composition and eating quality of sow meat and also compared with those of the commonly commercial pig breed-derived meat.

### Technological quality and proximate composition

The technological quality traits and proximate composition of pork LTL muscle samples of FP and CS are presented in Table 1. Regarding the technological quality, the pH values ranged from 5.67 to 5.71 without significant differences between the two pork groups (p = 0.5057). Data similar to our study for pH were reported by Pieszka et al [12] and Yoo et al [13] for pork loins of FP. In general, these ultimate pH values fell within the ranges (5.7 to 5.8) for the normal pork [14]. It has been shown that genotypes and stress (e.g., caused by fighting, beating and overcrowding and transportation etc.) imposed on pigs before slaughtering are the major causes affecting the muscle glycolytic rate and ultimate pH in meat [14]. Although the pig groups used in the present study were different in their genetics, however, from our obtained results it may be suggested that the glycolytic and pH fall rates were similar in the FP- and CS-derived LTL muscles. Significant differences were observed for cooking loss and WBSF between two pork groups. The WHC is defined as the ability of fresh meat to retain its water during cutting, processing, storage and cooking, and usually is described as drip and cooking loss etc. Results revealed that the CS meat exhibited lower (p = 0.0366) cooking loss (19.62%) together with higher (p = 0.0213) WHC (63.11%). Lean meat comprises approximately 75% water at rigor, with the majority (85%) of the water being held within the myofibrils and these occupy about 82% to 87% of the volume of the muscle [15]. Structurally, water loss from meat muscle has been shown to be influenced by a variety of the structural elements of the muscle such as, extent of shrinkage of myofibrils at the rigor and interfilament spacing, the permeability of the cell membrane, and the development of drip channels and extracellular space [16]. On the other hand, researchers have demonstrated that genetics and feeding diet are among the important intrinsic factors impacting the muscle characteristics which subsequently affect its WHC [17]. For instance, dietary supplementations with vitamin E, D_3_, or minerals improve the WHC of pork, and the mechanisms underlining this phenomenon is attributed to the improved muscle cell membrane stability resulting from reduced oxidation of lipids within the membrane [17]. Although pH is known as the most important factor affecting the WHC of meat [16], the pH values were similar in both pork groups in the present study. Therefore, the results indicating higher WHC with lower cooking loss in the CS meat could be due to the genetics and feeding regime effects (e.g., supplementations with minerals and vitamins in sow’s diets) which might result in improved stability of the muscle cell’s structural membrane as well as their WHC. Our results agree with the general findings that pork of CS possesses higher WHC and shear force value [3]. The CS meat exhibited significantly higher (p<0.0001) WBSF value (4.55 kgf/cm^3^) compared to the FP meat (2.85 kgf/cm^3^). This could be related to the higher collagen content (2.52%) in the sow meat compared to the FP meat (1.20%, data not shown). Nevertheless, compared with the shear force values (2.85 to 4.88 kgf/cm^3^) in the present study, Yoo et al [13] reported higher values (6.49 to 7.45 kgf) for *longissimus dorsi* muscle of 19-weeks old pigs.

Regarding proximate composition, all the contents such as moisture and protein (except the fat) differed significantly between the FP and CS meats. The CS meat exhibited higher (p = 0.0312) moisture content (72.00%) while the FP meat had higher (p = 0.0129) protein content (25.63%). The higher protein level in FP meat could be due to its lower moisture content. Otherwise, the difference in moisture content could be related to the age, genetics and feeding diet differences between the pig groups. Compared with our data, those of Lebret et al [18] showed higher moisture content (79.9% to 80.2%) and lower protein (18.2% to 18.5%) in pork loin from FP.

### Instrumental color traits

Table 2 shows the instrumental color traits of pork LTL muscle of FP and CS at 24 h *postmortem*. Differences were found for all the color traits (except for lightness) between the two pork groups. The a* (redness), b* (yellowness), and Chroma values (11.10, 5.66, and 12.49, respectively) for the CS meat were significantly higher than those of FP meat (6.06, 397, and 7.09, respectively). The results indicate a redder color for the CS meat, which agrees with finding of Sindelar et al [3]. The differences observed for the colors could be related to the physico-chemical components difference between the pork groups, for instance the myoglobin concentration has been reported to increase in sow pork [3]. Additionally, compared with the values of color traits (lightness, redness, and yellowness) of CS meat in the present study, these authors reported much higher values, probably due to the differences in slaughter ages and genetics of the animals used between the studies. According to a previous study [19], *longissimus* muscle of commercial FP had lower L* values (49.67 to 50.21) and higher a* values (15.99 to 16.33) at 24 h *post-mortem* compared with those of both CS and FP meats in the present study. Moreover, Pieszka et al [12] reported higher L* value (56.7), a* value (15.4), and b* value (5.95) for *longissimus lumborum* muscle of FP at 24 h *post-mortem* compared with those of current study. These contrasting results could be due to the differences in animal genetics, feeding diets and pre- and post-slaughter handling among the studies. Additionally, from the results/observations of our investigation it may be said that that the CS meat is not redder in color than the commercial pork studied in the above cited literatures.

### Fatty acid profiles

The relative percentage of fatty acids in the *longissimus dorsi* muscle samples of FP and CS are shown in Table 3. The outcome of our analysis revealed that among 14 fatty acids identified, levels of 5 fatty acids significantly differed between the FP and CS meats. Particularly, the CS meat exhibited higher contents of C18:2n-6 (25.47%, p = 0.0231), C18:3n-3 (0.46%, p = 0.0269), C20:4n-6 (7.87%, p = 0.0233) and C22:4n-6 (0.79%, p = 0.0073) than the FP meat. Oleic acid (C18:1) was the most predominant fatty acid in the FP (43.06%) and CS (36.08%) meat, but its levels did not differ between the two pork groups (p = 0.0856). The FP meat had higher C16:0 level (26.75%, p = 0.0198), which resulted in significantly higher content of total saturated fatty acids (SFA, 39.47%) in this pork type compared to the CS meat (26.56%). Interestingly, the CS meat exhibited higher total levels of unsaturated fatty acids (UFA, 73.44%, p = 0.0409), especially polyunsaturated fatty acids (PUFA, 34.72%, p = 0.0213). Furthermore, the CS meat had greater levels of total n-6 fatty acids due to its higher levels of C18:2n-6 and C20:4n-6, which led to a significantly higher n-6/n-3 fatty acids ratio (p = 0.0267) as well as PUFA/SFA ratio (p = 0.0438) compared to the FP meat.

It is established that the fatty acids composition in meat is greatly affected by slaughter weight, environmental and nutritional factors [7,20], and the metabolism of lipid stores during sow’s lactation [4]. Also, Enser et al [21] reported that the proportion of energy available for fat deposition in pigs increases during growth, which leads to the increased rate of *de novo* fatty acids synthesis. Therefore, the differences in the fatty acids composition between the FP and CS meats could be due to the above mentioned factors, and these results also suggest that the *de novo* synthesis of UFAs were greater in the CS than in the FP pigs. Furthermore, when compared with the fatty acids composition of CS meat in the present study, those of Pieszka et al [12] reported higher total SFA content (43.81%) but lower UFA level (56.18%) and PUFA (16.195) in *longissimus lumborum* muscle of FP.

Apart from being the major energy source in human body, fatty acids are important for many biological processes, especially n-3 PUFAs produce a lot of beneficial effects on health such as, reducing the risk of heart disease and stroke reductions [22]. In the present study, the levels of n-3 fatty acids such as C18:3n-3 and C20:5n-3 were higher in the CS meat compared to FP meat. According to the nutritional recommendations, the n-6/n-3 PUFA ratio in the human diet should not exceed 4.0 because a higher ratio is associated with an increased risk of cancer, while the PUFA/SFA ratio should be above 0.45 [23]. According to the outcome of our analysis, the FP and CS meats both exhibited an undesirable n-6/n-3 ratio being several times higher than the recommended value of less than 4.0. While, the value of PUFA/SFA ratio was above the recommended value of 0.45 for the CS meat (1.41), which was also higher than value (0.36) reported for *longissimus lumborum* muscle of FP by Pieszka et al [12] and also higher than values (0.36 to 0.53) reported for *longissimus dorsi* muscle of FP by Alonso et al [24]. Thus, it may be said that the meat of CS partly showed a “healthier” fatty acids profile as it possesses a higher total UFA content and a more favorable PUFA/SFA ratio than the meat of FP.

### Flavor compounds

The concentrations of volatile flavor compounds detected in the cooked LTL muscle of FP and CS are presented in Table 4. A total of fifty-six compounds was identified from the pork samples, and they were classified into 7 chemical groups including aldehydes (20), alcohols (4), ketones (3), hydrocarbons (19), pyrazines (3), sulfur-and nitrogen-containing compounds (4), and furans (3). It was observed that aldehyde is one of the most predominant flavor class in the cooked pork samples of both CS and FP. This finding agrees well with that of Ba et al [7] and Benet et al [25], who reported a large number of aldehydes with their quantities in cooked pork.

Among 20 identified aldehydes, the amounts of 6 com pounds (3-methyl-butanal, 2-methyl-butanal, hexanal, E,2-heptenal, benzaldehyde and E,2-octenal) showed significant differences between the CS and finishing pig meats. Among these aldehydes, 3-methyl-butanal and 2-methyl-butanal are known to arise from free amino acids while, hexanal, E,2-heptenal and E, 2-octenal are derived from linoleic acid (C18: n-6), and benzaldehyde is known to arise from linolenic acid (C18:3n-6) [26,27]. The amounts of 3-methyl-butanal and 2-methyl-butanal were significantly higher in the FP meat than the CS meat, probably related to its higher levels of free amino acids which participated in the Strecker degradation process during cooking [28]. Unfortunately, the free amino acids composition (known as flavor precursors) were not determined for the pork samples in the current study. Also, the amounts of all the three C18:3n-6 derived- aldehydes (hexanal, E,2-heptenal and E, 2-octenal) were significantly higher in the CS meat than the FP meat. This result could be related to the significantly higher C18:3n-6 content in the CS meat (Table 3). Researchers have reported that although hexanal contributes positively to meat flavor it may also produce undesirable flavors at higher concentrations [29]. Furthermore, hexanal has been found associated with warmed-over flavor of cooked pork [30], and partly contributes to rancid attributes of meat [31]. Lastly, the amount of benzaldehyde was also higher in the CS meat compared to that of FP meat, probably due to its higher C18:3n-3 content (Table 3). Regarding the total aldehydes content, the CS meat exhibited significantly (p = 0.0458) higher amount (1.25 μg/g) than the FP meat (1.02 μg/g). We observed that all the aldehydes (except for 3-methyl-butanal, 2-methyl-butanal) were formed by the oxidation of UFA during cooking, which agreed with the finding of Benet et al [25]. Furthermore, most of the aldehydes detected in our samples have also been identified and reported for cooked pork of FP by other authors [32].

Alcohols partly contribute to cooked meat flavors due to their low odor threshold [33]. Results showed that no differences were observed for all the identified alcohols as well as their total contents between two pork groups. Pyrazines are the Maillard reaction products which significantly contribute to the roast attributes of cooked meat [28,34]. No differences were found for all the pyrazines between the CS and FP meats. Regarding the sulfur-and nitrogen-containing compounds, it was observed that only Carbon disulfide was found in both pork groups whereas, two compounds (3-(methylthio)-propanal and 4-methylthiazole) were only found in the FP meat. 3-(methylthio)-propanal is formed from the breakdown of sulfur-containing amino acids via Strecker degradation, and 4-methylthiazole is known as the product of Maillard reaction [28]. These two compounds both play a key role in cooked meat flavor with the meaty and roast odor notes [35].

Regarding ketones, only 2-heptanone was found in both FP and CS meats while, 2,5-dimethyl-3-hexanone and 2,5-octanedione were detected in the FP and CS meat, respectively. 2-Heptanone is known to be formed from the oxidation of C18:2n-6 [26], and has been reported to confer fruity, spicy, cinnamon odor notes in beef [29]. Our result indicated a higher amount (0.12 μg/g) of 2-heptanone in the CS meat (p = 0.0178), which could be associated with its higher C18:2n-6 content (Table 3). Furans are known as the products produced either from the Maillard reaction between free amino acids and sugar or the oxidation of UFA [26,27]. All the identified furans (2-pentylfuran, 2-heptylfuran, and 2-octylfuran) showed differences between the two pork groups with significantly higher concentrations for the CS meat. Researchers have reported that 2-pentylfuran, and 2-heptylfuran and 2-octylfuran are the oxidation products of C18:2n-6 and C18:1n-6, respectively [26]. Furans seem to contribute less to the flavor characteristics of cooked meat due to their high odor-detection thresholds, and our obtained results may be attributed to the different contents of UFA between the pork groups (Table 3).

Hydrocarbons are known as the lipid oxidation-derived products which less contribute to the cooked meat flavors because of their high odor-detection thresholds [26,27]. Results showed that hydrocarbons were the second most predominant class, with 18 compounds being found in the pork samples in the present study. However, significant differences were observed only for 6 compounds (benzene, toluene, 1,3-dimethyl-benzene, decane, Z,3-dodecene, and nonanoic acid) between the meats of FP and CS.

Overall, it was also observed that the number and quantity of volatile flavor compounds in the cooked pork samples were considerably impacted by their animal origins (FP or sow). Typically, the lipid oxidation-derived products (e.g., aldehydes and hydrocarbons) rather than the other remaining Maillard reaction products (e.g., pyrazines, sulfur-and nitrogen-containing compounds) in the cooked pork samples were more affected by the pork type. We assume that the results indicating the differences in the number and quantity of flavor compounds could be attributed to the different levels of flavor precursors (e.g., fatty acids, free amino acids, and sugars) in the muscle tissues between the FP and CS. Therefore, further study may be needed to determine the levels of flavor precursors in the meats of these pigs. Additionally, further study in characterizing the odor/flavor characteristics of the detected volatile compounds by using particular techniques (e.g., gas chromatography with olfactometric detection) to elucidate which compounds associated with desirable or undesirable flavors in the meat samples is necessary.

### Sensory characteristics

The results of sensory evaluation of meats derived from FP and CS are presented in Table 5. On a 7-points scale, the sensory panel scores given by the panelists for color, flavor, juiciness and tenderness, were 4.52 vs 4.38, 3.99 vs 4.05, 3.44 vs 3.38, and 3.42 vs 3.43 for the FP and CS meat, respectively. However, no statistical differences were found for all these sensory attributes (color, flavor, juiciness, and tenderness) scores between the two pork groups. For the sensorial color, although the instrumental color analysis revealed a higher a* value (redness) in the CS meat (Table 2), the panelists did not find any differences in sensorial color scores (p = 0.4662). In fact, before using for the sensory evaluation, the pork samples were frozen at −20°C for approximately 2 months, which might result in the denaturation of myoglobin and hemoglobin molecules, and loss of optimum color presentation [36,37]. Thus, it may be suggested that the sow meat seemed to have lesser color stability compared to the commercial pork after freezing and thawing. Similarly, although a considerable variation in the volatile flavor compounds, especially the PUFA-derived compounds such as aldehydes and hydrocarbons were observed (Table 4), the panelists reported no significant differences in flavor scores between the pork groups (p = 0.6791). Also, although the CS meat inhibited higher shear force value (Table 1), no difference in tenderness scores were reported by the panelists for the two pork groups (p = 0.9310). Lastly, the overall acceptability scores given by the panelists were not significantly different between the two pork groups (p = 0.0532). In general, the panelists did not discriminate the sensory quality of commercial pork from the CS meat. These sensory evaluation results suggest that the eating quality of CS meat could be comparable to that of commercial pork.

## CONCLUSION

The present study for the first time determined the technological quality traits, fatty acids composition, flavor compounds and eating quality of pork from CS, and compared with those of commercial pork. There were large variations in the technological quality traits, chemical composition (e.g., fat content and fatty acids profile) and the contents of flavor compounds between meats of FP and CS. Typically, the meat of CS exhibited higher contents of total UFA and PUFA as well as more desirable PUFA/SFA ratio compared to those of commercial pork. A total of fifty-six flavor compounds was identified from the pork samples, with sixteen compounds showed significant differences between the two pork groups, and most of them were the oxidation products (e.g., aldehydes and hydrocarbons) of PUFA. Although the sow meat exhibited higher concentrations of some compounds (e.g., hexanal) that associated with undesirable flavors as well as higher shear force value, no differences were reported by the panelists for the flavor, tenderness and acceptability scores between two pork groups. From the sensory evaluation results obtained in the present study, it may be said that eating quality of CS meat could be comparable to that of commercial pork.

## Figures and Tables

**Table 1:** Technological quality traits and proximate composition of pork *longissimus thoracis et lumborum* muscle from cull sows and finishing pigs

Items	Finishing pork	Sow pork	p-values
pH	5.71±0.031)	5.67±0.05	0.5057
Water holding capacity (%)	60.99±0.95^b^	63.11±1.43^a^	0.0213
Cooking loss (%)	29.73±0.91^a^	19.62±4.88^b^	0.0366
WBSF (kgf)	2.85±0.08^b^	4.55±0.30^a^	<0.0001
Moisture (%)	70.43±0.45^b^	72.00±0.40^a^	0.0312
Fat (%)	4.53±0.33	4.57±0.26	0.9432
Protein (%)	25.63±0.86^a^	22.33±0.20^b^	0.0129

WBSF, Warner-Bratzler shear force.

1) Means±standard errors.

a,b Means within a same row with different letters are significantly different.

**Table 2:** Color traits of pork *longissimus thoracis et lumborum* muscle from cull sows and finishing pigs

Items	Finishing pork	Sow pork	p-values
L*(Lightness)	53.62±0.881)	51.50±1.13	0.1483
a* (Redness)	6.09±0.25^b^	11.10±0.67^a^	<0.0001
b* (Yellowness)	3.97±0.25^b^	5.66±0.46^a^	0.0010
Chroma	7.09±0.32^b^	12.49±0.78^a^	<0.0001
Hue angle	34.15±1.42^a^	26.57±1.09^b^	0.0008

1) Means±standard errors.

a,b Means within a same row with different letters are significantly different.

**Table 3:** Relative percentage (%) of fatty acids in pork *longissimus thoracis et lumborum* muscle from cull sows and finishing pigs

Items	Finishing pork	Sow pork	p-values
C14:0	1.55±0.101)	1.15±0.15	0.0868
C16:0	26.75±1.25^a^	21.27±0.76^b^	0.0198
C16:1n7	2.25±0.24	1.85±0.20	0.2758
C18:0	11.17±0.56	4.14±3.05	0.0856
C18:1n9	43.06±3.11	36.08±1.36	0.1084
C18:1n7	0.18±0.02	0.13±0.00	0.0732
C18:2n6	12.31±1.19^b^	25.47±3.48^a^	0.0231
C18:3n6	0.08±0.01	0.10±0.02	0.4100
C18:3n3	0.39±0.05^b^	0.46±0.08^a^	0.0269
C20:1n9	0.54±0.07	0.66±0.13	0.4321
C20:4n6	1.41±0.42^b^	7.87±1.76^a^	0.0233
C20:5n3	0.04±0.01	0.09±0.01	0.0501
C22:4n6	0.16±0.02^b^	0.79±0.12^a^	0.0073
C22:6n3	0.05±0.01	0.03±0.00	0.3377
SFA	39.47±1.86^a^	26.56±3.92^b^	0.0409
UFA	60.53±1.86^b^	73.44±3.92^a^	0.0409
MUFA	46.02±2.94	38.72±1.38	0.0876
PUFA	14.51±1.55^b^	34.72±5.28^a^	0.0213
n-3	0.55±0.08	0.49±0.08	0.5898
n-6	13.96±1.52^b^	34.23±5.24^a^	0.0206
n-6/n-3	25.98±3.28^b^	71.99±13.03^a^	0.0267
MUFA/SFA	1.18±0.13	1.50±0.15	0.1788
PUFA/SFA	0.37±0.04^b^	1.41±0.36^a^	0.0438

SFA, saturated fatty acids; UFA, unsaturated fatty acids; MUFA, mono unsaturated fatty acids; PUFA, poly unsaturated fatty acids.

1) Means±standard errors.

a,b Means within a same row with different letters are significantly different.

**Table 4:** Volatile aroma compounds (μg/g) of cooked pork *longissimus thoracis et lumborum* muscle from cull sows and finishing pigs

Items	Retention time (min)	Finishing pork	Sow pork	p-values	Identification method1)
Aldehydes					
2-methyl-propanal	1.936	0.01±0.002)	0.01±0.00	0.0556	MS+STD
3-methyl-butanal	2.697	0.02±0.01^a^	0.002±0.00^b^	0.0033	MS+STD
2-methyl-butanal	2.806	0.05±0.01^a^	0.01±0.00^b^	0.0058	MS+STD
Pentanal	3.181	0.01±0.00	0.002±0.00	0.1146	MS+STD
2-methyl-pentanal	3.281	0.04±0.01	0.05±0.00	0.6552	MS+STD
Hexanal	6.085	0.50±0.10^b^	0.73±0.02^a^	0.0416	MS+STD
Heptanal	9.260	0.04±0.01	0.05±0.01	0.5701	MS+STD
(E), 2-Heptenal	10.748	0.04±0.00^b^	0.06±0.00^a^	0.0009	MS+STD
Benzaldehyde	10.868	0.11±0.00^b^	0.14±0.01^a^	0.0037	MS+STD
Octanal	11.915	0.07±0.02	0.11±0.02	0.1452	MS+STD
Benzene acetaldehyde	12.876	0.01±0.00	nd	-	MS
(E),2-octenal	13.189	0.002±0.00^b^	0.01±0.00^a^	0.0018	MS+STD
(E), 2-Decenal	13.981	0.03±0.02	0.02±0.00	0.6472	MS+STD
Nonanal	14.198	0.09±0.02	0.07±0.02	0.5550	MS+STD
Decanal	16.229	0.01±0.00	0.01±0.00	0.8472	MS+STD
(E,E), 2,4-Decadienal	18.295	0.01±0.00	0.01±0.00	0.1402	MS+STD
2-Undecenal	18.999	0.004±0.00	nd	-	MS
E-2-decenal	19.075	0.003±0.00	nd	-	MS
Dodecanal	19.823	0.002±0.00	0.003±0.00	0.0929	MS+STD
Tridecanal	21.677	0.001±0.00	0.001±0.00	0.1825	MS+STD
Total aldehydes		1.02±0.16^b^	1.25±0.05^a^	0.0458	
Alcohols					
1-Pentanol	5.026	0.01±0.002)	0.01±0.00	0.5220	MS+STD
1-Heptanol	11.103	0.03±0.00	0.04±0.00	0.0963	MS+STD
1-Octen-3-ol	11.343	0.02±0.00	0.04±0.00	0.2326	MS+STD
2-ethyl-1-hexanol	12.516	nd	0.01±0.00	-	MS
Total alcohols		0.06±0.01	0.07±0.01	0.4646	
Pyrazines					
2,5-dimethyl-pyrazine	9.558	0.01±0.00	0.01±0.00	0.8533	MS+STD
2,6-dimethyl pyrazine	9.690	0.01±0.00	0.01±0.00	0.7396	MS+STD
2-ethyl-3,5-dimethyl-pyrazine	13.575	0.01±0.00	0.01±0.00	0.1572	MS
Total pyrazines		0.03±0.00	0.03±0.00	0.1014	
Sulfur-and nitrogen-containing compounds					
Carbon disulfide	1.862	0.01±0.00^a^	0.001±0.00^b^	0.0435	MS
3-(methylthio)-propanal	9.391	0.01±0.00	nd	-	MS+STD
4-Methylthiazole	11.423	0.16±0.02	nd	-	MS+STD
1-ethyl-2(1H)-Pyridinone	16.602	nd	0.01±0.00	-	MS
Total sulfur-and nitrogen-compounds		0.17±0.02^a^	0.02±0.00^b^	<0.0001	
Ketones					
2-heptanone	8.911	0.03±0.01^b^	0.12±0.03^a^	0.0178	MS+STD
2,5-dimethyl-3-hexanone	10.554	nd	0.09±0.00	-	MS
2,5-octanedione	11.472	0.12±0.01	nd	-	MS+STD
Total ketone		0.15±0.02^b^	0.21±0.04^a^	0.0102	
Furans					
2-pentyl furan	11.581	0.11±0.01^b^	0.73±0.09^a^	<0.0001	MS+STD
2-n-Heptylfuran	15.812	0.01±0.00^b^	0.04±0.00^a^	0.0047	MS+STD
2-n-Octylfuran	17.889	0.04±0.00^b^	0.08±0.01^a^	<0.0001	MS+STD
Total furans		0.15±0.02^b^	0.84±0.01^a^	<0.0001	
Hydrocarbons					
Bezene	1.690	0.04±0.00^a^	0.01±0.00^b^	0.0278	MS
2-Propenoic acid	2.136	0.02±0.00	0.01±0.00	0.0571	MS
Toluene	4.929	0.03±0.00^a^	0.01±0.00^b^	0.0097	MS+STD
Butanoic acid	5.591	0.01±0.00	nd	-	MS+STD
1,3-dimethyl-benzene	8.299	0.03±0.02^b^	0.21±0.06^a^	0.0041	MS
Hexanoic acid	11.264	0.01±0.00	nd	-	MS
Decane	11.847	0.02±0.00^b^	0.05±0.00^a^	0.0002	MS
3-ethyl-2-methyl-1,3-hexadiene	12.562	0.01±0.00	nd	-	MS
2,2-dimethyl heptane	13.034	0.01±0.00	0.01±0.00	0.1892	MS
2,2,6-trimethyl octane	13.290	0.02±0.00	0.003±0.00	0.2716	MS
Z,3-Dodecene	14.765	0.05±0.01^b^	0.15±0.04^a^	0.0103	MS
Benzoic acid	15.450	0.11±0.07	0.18±0.06	0.4708	MS
Octanoic acid	15.534	0.17±0.10	0.01±0.00	0.1878	MS
1-phenyl 1,2-Propanedione	15.932	0.03±0.01	0.03±0.00	0.9512	MS
E-1,9-Dodecadiene	16.098	0.12±0.06	0.01±0.00	0.1314	MS
Nonanoic acid	17.271	0.10±0.03^a^	0.01±0.00^b^	0.0104	MS
2-methyl-undecane	17.471	0.04±0.01	0.08±0.01	0.2518	MS
3,4-dimethyl heptane	18.190	0.06±0.02	0.17±0.01	0.2602	MS
3,7-dimethyl decane	18.614	0.12±0.06	0.34±0.03	0.4392	MS
Total hydrocarbons		1.04±0.02	1.13±0.08	0.3187	MS

nd, not detected; -, insufficient data for calculation.

1) Identification method: The compounds were identified by either mass spectra (MS) from library or authentic standards (STD).

2) Means±standard errors.

a,b Means within a same row with different letters differ significantly.

**Table 5:** Sensorial evaluation scores (7-points scale) of cooked pork *longissimus thoracis et lumborum* muscle from cull sows and finishing pigs

Items	Finishing pork	Sow pork	p-values
Color	4.52±0.121)	4.38±0.14	0.4662
Flavor	3.99±0.10	4.05±0.09	0.6791
Juiciness	3.44±0.10	3.38±0.10	0.6781
Tenderness	3.42±0.12	3.43±0.13	0.9310
Acceptability	3.38±0.08	3.63±0.09	0.0532

The mean values were calculated using 7-point scale (7 = extremely like; 6 = like very much; 5 = like moderately; 4 = neither like nor dislike; 3 = dislike moderately; 2 = dislike very much; and 1 = dislike extremely).

1) Means±standard errors.
